# Supplementary figures and images for: Olive Leaf Tea Impact on Postprandial Glycemia: A Randomized Cross-Over Trial
Source: Foods. 2023 Jan 24;12(3):528. doi: 10.3390/foods12030528 (PMC9914386; doi:10.3390/foods12030528)

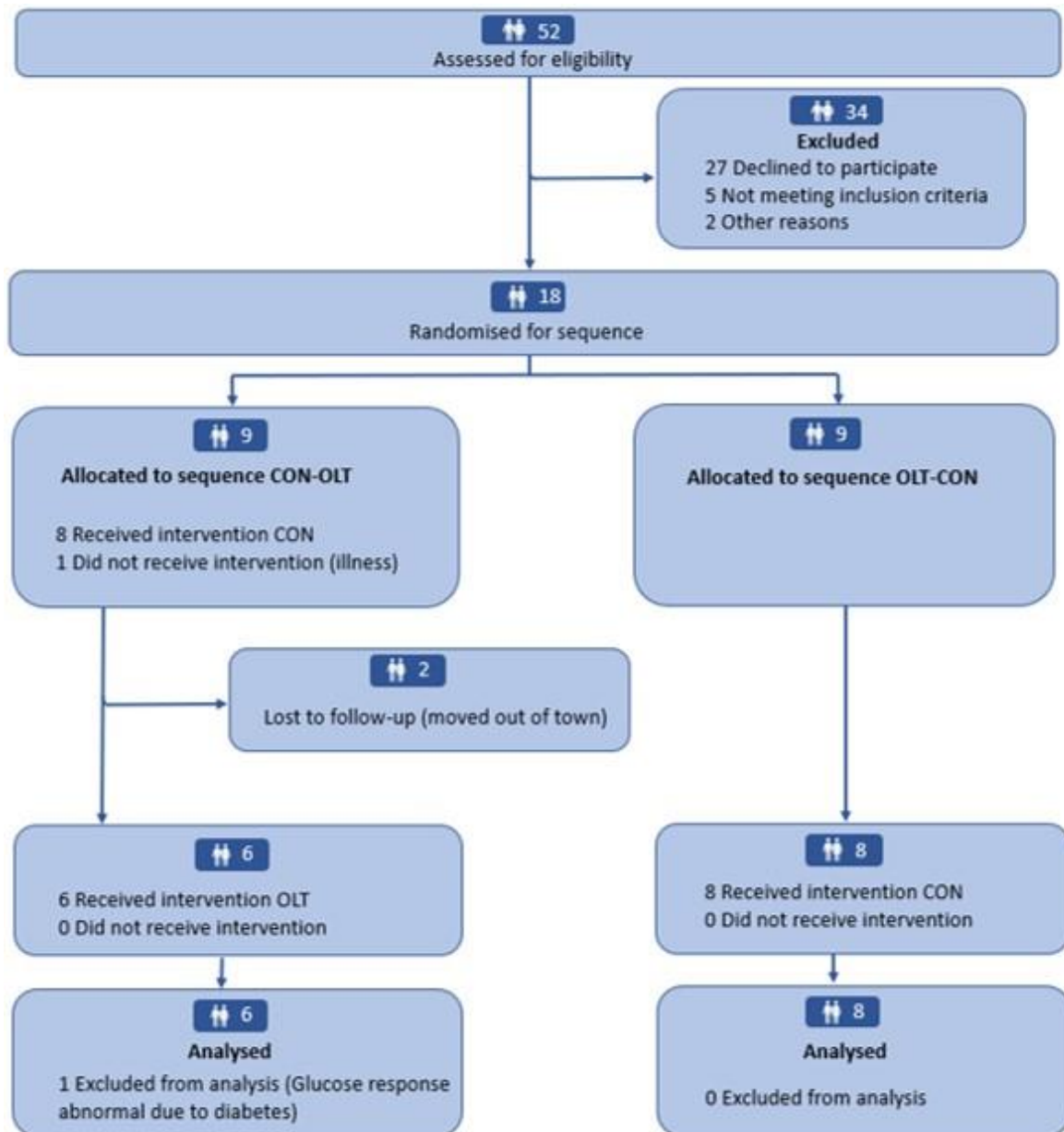

Figure S1. CONSORT flowchart of the trial flow.

Supplement: Supplementary file 1 [file foods-12-00528-s001.zip › foods-2134165-supplementary.pdf]
